# Palate & Stomach

**Published:** 1875-10

**Authors:** 


### THE PALATE AND THE STOMACH.

A good lady whom we met on a long journey, declared that food eaten by her at a dingy railroad station, amid unpleasant and uncomfortable surroundings, never did her any good; was devoid of nourishment, and might better be left untouched, for all the strength it imparted. Hundreds of times we have observed, as have all medical men, that a patient kept unaccountably weak, long after convalescence should be attended with growing strength. When such a condition exists, we make it a point to stop to dinner. The secret of the trouble, nine times in ten, is in the food. It isn't palatable; it tastes unpleasantly, even; it has acquired a bad and unwholesome flavor from unclean pots and kettles, or it is mixed with unsavory greases, or it is burned or ruined in some of the many ways by which good food can be spoiled. We set these essential conditions aright, and our patient eats for strength,—eats and lives. What we mean is, that the stomach and the palate sympathize very closely. This can scarcely be otherwise. The stomach is only a continuation of the mouth. One membrane of like character covers the whole. You can not impose upon the palate, without a demurer on the part of the stomach. The stomach is more watchful than you think; the instant the sense of taste is offended by some disagreeable and nauseous mouthful, the stomach recoils and exclaims: "An offender approaches; I will treat him violently; I will expel him!" Thousands there be who go through life with a profound disgust at some good and wholesome article of food, because in childhood it was in some way associated with sickness. One can not bear gruel, because in one of the diseases of childhood it was his sick-bed diet. We ourselves confess to a life-long, incurable repugnance to baked apples—a most wholesome article of food—because in early life they were made to conceal and disguise the horrid pill, which our good mother periodically forced down our unwilling throat. It was too bad, thus to destroy forever our desire for a good, palatable food.

It is important to make medicines as nearly tasteless as possible, if this can be done without destroying their potency. This fact has been so evident, that the highest resources of the chemist's art are drawn upon to conceal nauseous flavors.

No method succeeds which merely disguises and does not wholly conceal disagreeable flavors. Dr. Dundas Dick, the distinguished chemist, has a method by which bad tasting medicaments are conveyed into the stomach, without the least disturbance to the stomach. He does not mix his potent oils and balsams with some absorbent, and so create a pill to be swallowed with only a partial sense of misery. He encloses his fluids in a capsule, a film of pure gelatine, and seals it up. The medicine thus securely sealed, is forever protected from deterioration, and is ready for use at all times. The little oblong sphere with its potent contents is readily swallowed, and is quickly dissolved in the fluids of the stomach. The palate is spared all suffering, and the stomach is not a little appeased, because its sensitive neighbor has not been offended.

There is hardly any potent fluid of unpleasant taste, which may not thus be protected in its passage to the stomach. The use of medicines thus prepared is rapidly extending, and new articles are being added to the list. Of course, the very fact of this concealment renders it possible for dishonest or incompetent men to thus enclose in bottles of gelatine, bad and impure drops. This would be a cruel deceit; but medicines do not escape this form of wickedness. The only safety in this, as in other things, is to buy only the products of persons of repute. Happily there are reputations honorable and dishonorable gained in the walks of trade, and to these we must look when a question arises. We are happy to say, that Dr. Dick's reputation is of the best, and to express our conviction that his name is a guarantee of the honesty and purity of his products. We employ his remedies,—which are not secret preparations, but simply well-known articles of our materia medica, protected as we have described,—with great satisfaction, and hear only good accounts from their use.

